# Combat Injury, Limb Loss, Traumatic Brain Injury, and Psychosocial Outcomes Among Military Personnel

**DOI:** 10.1001/jamanetworkopen.2026.37592

**Published:** 2026-10-06

**Authors:** Daniel Dyball, Cheryl R. Z. See, Alexander N. Bennett, Susie Schofield, Grace Blissitt, Lydia Orton, Harriet Kemp, Christopher J. Boos, Anthony M. J. Bull, David J. Sharp, Neil S. N. Graham, Simon Wessely, Howard Burdett, Nicola T. Fear

**Affiliations:** 1 King’s Centre for Military Health Research, King’s College London, London, United Kingdom; 2 Department of Brain Sciences, Imperial College London, London, United Kingdom; 3 UK Dementia Research Institute Centre for Care Research & Technology, Imperial College London and University of Surrey, London, United Kingdom; 4 Academic Department of Military Rehabilitation, Defence Medical Rehabilitation Centre, Stanford Hall Estate, Nottinghamshire, United Kingdom; 5 National Heart and Lung Institute, Faculty of Medicine, Imperial College London, London, United Kingdom; 6 Department of Surgery and Cancer, Imperial College London, London, United Kingdom; 7 Faculty of Health & Social Sciences, Bournemouth University, Bournemouth, United Kingdom; 8 Centre for Injury Studies, Department of Bioengineering, Imperial College London, London, United Kingdom; 9 Academic Department of Military Mental Health, King’s College London, London, United Kingdom

## Abstract

**Importance:**

Given the burden and rising number of injuries from recent conflict zones such as Ukraine and the Middle East, understanding the long-term health outcomes of those with physical traumatic injuries is critical.

**Objective:**

To investigate psychosocial outcomes among UK military personnel who sustained physical combat injuries during deployments to Afghanistan and how traumatic amputation and traumatic brain injury (TBI) are associated with these outcomes.

**Design, Setting, and Participants:**

This cohort study used data from a prospective cohort (Armed Services Trauma and Rehabilitation Outcome [ADVANCE]) that includes a group of participants who sustained physical combat injuries and a frequency-matched, demographically similar uninjured group. Data were assessed a median of 11 (IQR, 10-13) years following injury at the UK Defense Medical Rehabilitation Centre (Stanford Hall) from August 2018 to August 2023. Data were analyzed from March 2024 to July 2026.

**Exposures:**

Physical combat injury, including injury profiles with and without limb loss or with deployment-related TBI of varying severity.

**Main Outcomes and Measures:**

Psychometrically validated self-report questionnaire data indicating probable alcohol misuse, generalized anxiety, depression, posttraumatic stress disorder (PTSD), or mental health multimorbidity (MHM).

**Results:**

A total of 1047 participants were included in the analysis (median age, 37 [IQR, 34-41] years; 100% male). A total of 523 had sustained combat injuries (141 with limb loss and 382 without, 225 with any TBI, 144 with mild TBI, and 81 with moderate to severe TBI), and 524 were in the uninjured group. Among nonofficers who sustained physical combat injuries, increases in the risk of reporting probable generalized anxiety (adjusted risk ratio [ARR], 1.92 [95% CI], 1.40-2.95]), depression (ARR, 1.78 [95% CI, 1.37-2.44]), PTSD (ARR, 2.00 [95% CI, 1.39-3.00]), and mental health multimorbidity (ARR, 2.05 [95% CI, 1.35-3.16]) were observed compared with participants who were not injured. Limb loss resulted in only small or inconclusive differences in the risk of reporting any mental health outcomes (lowest ARR for generalized anxiety, 1.24 [95% CI, 0.65-2.15]; highest ARR for MHM, 1.36 [95% CI, 0.63-2.59]), whereas greater risk was observed in those injured without limb loss (lowest ARR for depression, 1.95 [95% CI, 1.47-2.67]; highest ARR for MHM, 2.32 [95% CI, 1.52-3.60]). Injuries with mild TBI also resulted in greater risk (lowest ARR for depression, 2.31 [95% CI, 1.63-3.29]; highest ARR for MHM, 2.63 [95% CI, 1.62-4.80]), whereas moderate to severe TBI resulted in smaller elevations with greater uncertainty (lowest ARR for generalized anxiety, 1.52 [95% CI, 0.72-2.78]; highest ARR for MHM, 2.25 [95% CI, 1.11-4.21]). Co-occurring TBI increased observed risks in those injured with and without associated limb loss. All injury types investigated were associated with only small or inconclusive risks for reporting alcohol misuse.

**Conclusions and Relevance:**

Specific injury profiles, namely deployment-related TBI or injuries without limb loss, were associated with elevated risk of poor mental health outcomes among UK military personnel. Injury profiles may be a key contextual factor in physical combat injury research for the identification of individuals at elevated risk of poor mental health outcomes.

## Key Points

**Question:**

What are the long-term associations between sustaining a physical combat injury during deployments to Afghanistan and mental health outcomes among military personnel in the UK?

**Findings:**

In this cohort study, which used data from the Armed Services Trauma and Rehabilitation Outcome sample of 1047 participants, injured personnel had increased risks of reporting generalized anxiety, depression, posttraumatic stress disorder, and mental health multimorbidity compared with uninjured personnel. The greatest risk was observed in specific injury profiles, namely deployment-related traumatic brain injury or injuries without limb loss; however, injuries with limb loss resulted in only small and/or inconclusive differences in risk.

**Meaning:**

These findings suggest that military health care policy should afford comparable recognition and support to nonvisible combat-related injuries, including traumatic brain injury, alongside visible injuries such as limb loss.

## Teaser

This cohort study assesses the risk of poor psychosocial outcomes among UK military personnel with physical combat injury compared with deployed personnel without injury.

## Introduction

Given recent escalation of armed conflicts in Ukraine and the Middle East, the number of individuals sustaining physical injuries or complex multiple trauma has increased.^1,2^ Advances in medical and defense technology during the military deployments to Afghanistan (2002-2014) meant that UK military personnel were more likely to survive serious injuries that may have resulted in death compared with previous deployments to Iraq or Northern Ireland.^3^ Many of those injured during deployment to Afghanistan or Iraq experienced serious health sequelae, such as limb amputations or neurologic conditions that affect their physical and psychosocial functioning.^4^

The Armed Services Trauma and Rehabilitation Outcome (ADVANCE) cohort was established to investigate the long-term impact of sustaining a physical combat injury during deployment to Afghanistan, defined as injuries that required aeromedical evacuation to a UK hospital, on the health and well-being of UK military personnel.^4^ The initial mental health findings from this cohort suggested that sustaining a physical combat injury was associated with greater odds of reporting poor mental health outcomes compared with individuals who were demographically similar, but did not sustain physical combat injuries (uninjured group).^5^ However, while those who sustained injuries without any associated limb loss had greater odds of reporting poor mental health outcomes, those injured with limb loss did not. It is unclear why these injury profiles have divergent likelihoods of reporting poor mental health outcomes, although one possible explanation might be concurrent injuries, such as traumatic brain injury (TBI).^6^

TBI is associated with increased risk of common mental disorders (CMDs) and posttraumatic stress disorder (PTSD).^7^ However, evidence of associations between TBI and mental health is often based on TBI without stratification by severity.^7^ The Mayo Head Injury Classification System can be used to categorize the severity of TBI, including symptomatic, mild, and moderate to severe.^8^ While mild TBI has consistently been observed to be associated with increased risk of mental illness, there is mixed evidence for such an association for individuals with moderate to severe TBI.^7^ Given the lack of evidence for a clear dose-response relationship, further scrutiny of the levels of TBI severity is recommended.

This study used data from the first follow-up assessment of the ADVANCE cohort, a median of 11 (IQR, 10-13) years after the injury or the deployment of interest. UK military personnel with physical combat injuries are hypothesized to have an increased risk of poor psychosocial outcomes (alcohol misuse, CMDs [including generalized anxiety and depression], PTSD, or mental health multimorbidity [MHM]) compared with demographically similar uninjured personnel. This risk is expected to differ based on injury characteristics: personnel injured without limb loss and those with deployment-related TBI are hypothesized to have a greater risk of reporting poor psychosocial outcomes, whereas personnel with limb loss are hypothesized to have risk comparable with that of uninjured personnel.

## Methods

### Study Design and Participants

Full details of the ADVANCE study protocol^4^ and first follow-up assessment^9^ are available elsewhere. The injured group (n = 579) was recruited from a sample of UK Armed Forces personnel who were deployed to Afghanistan (Operation Herrick) between 2003 and 2014 and sustained physical combat injuries requiring aeromedical evacuation to a UK hospital. The uninjured group (n = 566) was recruited from individuals who were frequency matched to the injured group at the time of selection based on their age, rank, regiment, deployment era, and role during deployment. Participants attended a baseline appointment between 2015 and 2020. This cohort study reports on data from the first follow-up appointment, which occurred between August 2018 and August 2023, with median time since sampling injury or deployment of 11 (IQR, 10-13) years (eFigure in Supplement 1).

This study followed the Strengthening the Reporting of Observational Studies in Epidemiology (STROBE) reporting guideline for cohort studies. The ADVANCE Study has full ethical approval from the UK Ministry of Defense Research Ethics Committee. All participants provided written informed consent.

### Procedure

Participants were contacted through postal, email, and telephone invitations, having previously consented to take part. The study day occurred at the Defense Medical Rehabilitation Centre (Stanford Hall) and included medical assessments, a clinical interview, and a self-report questionnaire.

### Measures

Probable alcohol misuse, generalized anxiety, depression, and PTSD were measured using psychometrically validated self-report questionnaires and cutoff scores, and MHM was defined as probable PTSD alongside probable generalized anxiety and/or depression.^10,11,12,13,14^ Measures and associated cutoffs can be found in eTable 1 in Supplement 1.

Information on combat injury during deployment to Afghanistan (hereinafter referred to as index injury) was collected from electronic medical records and supplemented by medical history from the clinical interviews. Participants with isolated partial amputations (toe, partial foot, finger, and/or partial hand) without limb loss were coded as having nonamputation injuries. The Ohio State University Traumatic Brain Injury Identification Method was administered during the clinical interviews.^15^ This was cross-referenced with the Joint Theatre Trauma Register and medical history to identify deployment-related TBI (C.R.Z.S., G.B., L.O., et al, unpublished data, September 2025). Mayo classification was used to ascertain TBI severity.^8^ Briefly, this included (1) symptomatic (possible) TBI, defined as reports of TBI-related symptoms (eg, dazed, dizziness, headaches); (2) mild (probable) TBI, defined as any loss of consciousness ranging from momentary to 30 minutes, posttraumatic amnesia ranging from momentary to less than 24 hours, a Glasgow Coma Scale score of 13 to 15, or any reports of depressed, basilar, or linear skull fractures with dura intact; and (3) moderate to severe (definite) TBI, defined as loss of consciousness greater 30 minutes, posttraumatic amnesia lasting longer than 24 hours, a Glasgow Coma Scale score of 12 or less, or abnormal results of structural neuroimaging. Clinical magnetic resonance imaging scans were assessed by a consultant neuroradiologist to inform this decision. A binary variable capturing any deployment-related TBI was included and defined as any indication of mild or moderate to severe TBI, excluding symptomatic TBI (eTable 2 in Supplement 1).

Additional data collected included age at follow-up assessment (in years); ethnicity self-reported during the clinical interview with a binary code derived from these data to represent White and ethnic minority groups (categories included Bangladeshi, Black African, Black Caribbean, Chinese, Indian, Pakistani, Other Asian, and other or multiethnic group [excluding White minority groups]), in line with UK government classifications; serving status categorized as serving in a regular or reservist position at point of assessment; and rank at point of sampling used as a proxy for socioeconomic status.^16^ Ethnicity data were included because individuals with ethnic minority status have different associated risks with the primary hypotheses for the ADVANCE cohort (specifically cardiovascular disease), but also for psychosocial outcomes. Participants were coded as other rank or junior noncommissioned officer (NATO rank OR2-OR4), senior noncommissioned officer (NATO OR5-OR9), and officer rank (OF1-OF9).

### Statistical Analysis

Data were analyzed from March 2024 to July 2026. For data analysis, we used the statistical software package StataNow, version 19.0 (StataCorp LLC). All models controlled for socioeconomic status at sampling and age at follow-up assessment.^17,18^ A longitudinal weight for the first follow-up was established by multiplying an attrition weight by an overall baseline weight (eMethods in Supplement 1); assessment of attrition or loss to follow-up has been published previously.^9^ Weighted percentages are presented alongside unweighted cell counts in demographic tables only. Deployment-related TBI groups were coded as per eTable 2 in Supplement 1.

Generalized linear model regression analysis with a log-link was conducted to test all hypotheses. Symptomatic TBI was not individually reported in these models due to the lower level of certainty associated with this classification^19^; however, this group was retained as a categorical value for models investigating mild and moderate to severe TBI to maximize information and ensure conservative classification.^8^ In line with contemporary recommendations on statistical inference, adjusted risk ratios (ARRs) are presented alongside bias-corrected 95% CIs that were bootstrapped using 1000 replications, which were interpreted with attention to effect sizes, uncertainty, and broader context.^20,21^

One participant experienced a serious injury outside military service and another participant disclosed a health condition that was present prior to sampled deployment in line with exclusion criteria for the ADVANCE cohort study. Four participants did not complete any of the mental health questionnaires. These individuals were excluded from current analyses. A small number of individuals did not complete single measures such as the 7-item Generalized Anxiety Disorder scale (GAD-7) (n = 1) or the Posttraumatic Stress Disorder Checklist for *DSM-5* (PCL-5) (n = 2), which was handled using casewise deletion. Item level missingness was low (maximum of 1 item; GAD-7 [n = 1], Patient Health Questionnaire–9 [n = 1], and PCL-5 [n = 5]), which was handled using 2-way imputation of standard errors. Officers were excluded from regression analyses only, due to the small number of poor psychosocial outcomes reported in this group (range from outcomes investigated, 4-11).

## Results

### Demographics

Of the original baseline sample of 1145 participants, 1053 (92.0%) were retained for the first ADVANCE follow-up, of whom 1047 were included as part of this analysis (949 [90.4%] White and 98 [9.6%] other ethnic group; 100% male). Participants attended their first follow-up appointment a median of 11 (IQR, 10-13) years following their injury or deployment of interest. Participants’ median age at the follow-up appointment was 37 (IQR, 34-41) years, and they were predominantly other rank or junior noncommissioned officers at point of sampling (916 [92.6%]). More participants had left service at the point of assessment (582 [57.4%]) than were still serving in the armed forces (465 [42.6%]) (Table). The uninjured group included 524 participants, and the overall injured group included 523 (of whom 141 formed the amputation injury subgroup and 382 formed the nonamputation injury subgroup). Of the injured group, 225 participants sustained a deployment-related TBI (144 with mild TBI, and 81 with moderate to severe TBI). A deployment-related TBI was experienced by 72 (49.0%) of the amputation injury subgroup and 153 (39.9%) of the nonamputation injury subgroup (Figure 1).

**Table.** Sociodemographic and Deployment Characteristics at Follow-Up by Injury Status

Characteristic	Uninjured group overall (n = 524)	All combat injuries overall (n = 523)	Limb loss stratification		Deployment-related TBI by severity			
			Nonamputation injury (n = 382)	Amputation injury (n = 141)	None (n = 218)	Symptomatic (n = 80)	Mild (n = 144)	Moderate to severe (n = 81)
Age at sampled deployment, median (IQR), y	26 (23-29)	25 (22-29)	25 (22-29)	25 (22-28)	25 (22-29)	24 (22-27)	26 (22-29)	25 (22-30)
Age at follow-up assessment, median (IQR), y	38 (34-41)	37 (34-41)	37 (34-42)	37 (34-40)	37 (34-41)	36.5 (34-40)	38 (34-42)	37 (34-42)
Time from sampling to assessment, median (IQR), y^a^	11 (10-13)	12 (10-13)	12 (10-13)	11 (10-12)	11 (10-13)	11 (10-13)	12 (10-13)	12 (10-13)
Ethnicity, No. (weighted %)								
White	474 (90.9)	472 (90.1)	344 (89.9)	128 (90.6)	194 (88.9)	73 (90.9)	132 (91.1)	73 (90.8)
All other ethnic groups^b^	47 (9.1)	51 (9.9)	38 (10.1)	13 (9.4)	24 (11.1)	7 (9.1)	12 (8.9)	8 (9.2)
Left service at follow-up assessment, No. (weighted %)	168 (32.2)	414 (78.9)	276 (72.8)	NA (>90.0)^c^	154 (70.8)	69 (87.3)	119 (82.3)	72 (87.3)
Rank at sampled deployment, No. (weighted %)								
Other rank or junior noncommissioned officer	308 (66.7)	366 (76.2)	254 (73.3)	112 (84.9)	148 (73.8)	61 (83.7)	96 (72.9)	61 (81.9)
Senior noncommissioned officer	142 (24.7)	100 (17.4)	83 (19.8)	17 (10.1)	46 (19.6)	9 (9.1)	33 (21.2)	12 (12.3)
Officer	74 (8.6)	57 (6.4)	45 (6.9)	12 (5.0)	24 (6.6)	10 (7.2)	15(5.9)	8 (5.8)
Limb loss, No. (weighted %)								
Injured without limb loss	0	382 (74.9)	0	0	176 (81.2)	53 (68.4)	101 (72.8)	52 (67.1)
Injured with limb loss	0	141 (25.1)	382 (100.0)	141 (100.0)	42 (18.8)	27 (31.6)	43 (27.2)	29 (32.9)
Deployment-related TBI, No. (weighted %)								
None	524 (100)	218 (42.6)	176 (46.2)	42 (31.8)	218 (100)	0	0	0
Symptomatic^d^	0	80 (15.2)	53 (13.9)	27 (19.1)	0	80 (100)	0	0
Mild^e^	0	144 (27.3)	101 (26.6)	43 (29.5)	0	0	144 (100)	0
Moderate to severe^f^	0	81 (14.9)	52 (13.4)	29 (19.5)	0	0	0	81 (100)
Mental health outcomes, No. (weighted %) [95% CI]								
Alcohol misuse (AUDIT score ≥16)	34 (7.0) [5.0-9.8]	37 (7.5 (5.4-10.3]	29 (8.3) [5.7-11.8]	8 (5.3) [2.6-10.5]	16 (7.7) [4.7-12.5]	5 (5.7) [2.3-13.4]	13 (10.5) [6.1-17.5]	NA^c^
Anxiety (GAD-7 score ≥10)	46 (9.8) [7.4-12.9]	89 (18.5) [15.2-22.3]	73 (20.6) [16.6-25.3]	16 (12.0) [7.4-19.0]	39 (19.0) [14.1-25.2]	7 (9.9) [4.5-20.3]	32 (25.1) [18.2-33.5]	11 (13.5) [7.4-23.3]
Depression (PHQ-9 score ≥10)	66 (14.0) [11.0-17.5]	118 (23.7) [20.1-27.8]	94 (25.8) [21.5-30.7]	24 (17.4) [11.8-25.0]	50 (23.4) [18.0-29.8]	11 (14.6) [7.9-25.6]	41 (31.5) [24.0-40.2]	16 (19.7) [12.2-30.4]
PTSD (PCL-5 ≥38)	39 (8.4) [6.1-11.4]	80 (17.2) [14.0-21.0]	66 (19.4) [15.4-24.0]	14 (10.8) [6.4-17.7]	31 (16.0) [11.4-22.0]	9 (12.8) [6.5-23.8]	28 (22.7) [16.0-31.1]	12 (15.4) [8.7-25.7]
Mental health multimorbidity (caseness on PCL-5 and PHQ-9 or GAD-7)	33 (7.2) [5.1-10.1]	70 (14.9) [11.9-18.5]	57 (16.5) [12.8-20.9]	13 (10.2) [5.9-17.0]	29 (14.5) [10.2-20.3]	5 (7.6) [3.0-18.0]	24 (19.4) [13.3-27.5]	12 (15.3) [8.7-25.5]

Abbreviations: AUDIT, Alcohol Use Disorder Identification Toolkit; GAD-7, 7-item Generalized Anxiety Disorder; NA, not available; PCL-5 Posttraumatic Stress Disorder Checklist for *DSM-5*; PHQ-9, Patient Health Questionnaire–9; PTSD, posttraumatic stress disorder; TBI, traumatic brain injury.

^a^ Calculated from the difference between age at index injury for injured personnel or age at sampled deployment plus 0.5 (to reflect mean age during sampled year) for the uninjured group and age at baseline or follow-up assessment.

^b^ Includes Bangladeshi, Black African, Black Caribbean, Chinese, Indian, Pakistani, Other Asian, and other or multiethnic group.

^c^ Some data were suppressed to allow for confidentiality in line with the rounding policy of the UK Ministry of Defence.^22^

^d^ Defined as reports of TBI-related symptoms (eg, dazed, dizziness, headaches).

^e^ Defined as any loss of consciousness ranging from momentary to 30 minutes, posttraumatic amnesia ranging from momentary to within 24 hours, a Glasgow Coma Scale score of 13 to 15, or any reports of depressed, basilar, or linear skull fractures with dura intact.

^f^ Defined as loss of consciousness for longer than 30 minutes, posttraumatic amnesia for longer than 24 hours, a Glasgow Coma Scale score of 12 or less, or abnormal structural neuroimaging.

**Figure 1.**
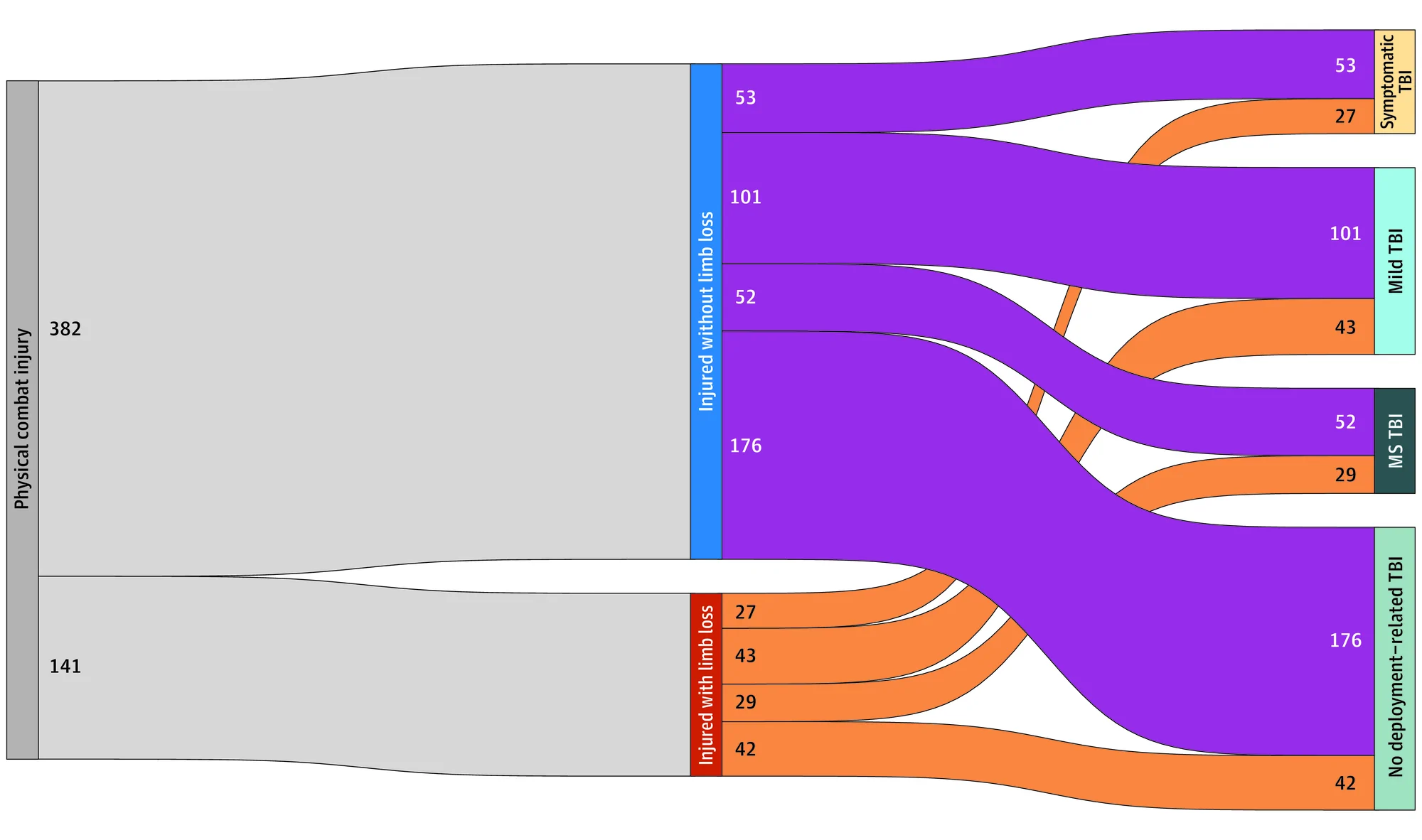
Sankey Diagram of Participants With Limb Loss and Deployment-Related Traumatic Brain Injury (TBI) MS indicates moderate to severe.

### Mental Health Outcomes

In the entire cohort, probable generalized anxiety was reported by 135 participants (14.5% [95% CI, 12.3%-16.9%]), depression by 184 (19.2% [95% CI, 16.8%-21.9%]), PTSD by 119 (13.1% [95% CI, 11.0%-15.5%]), and MHM by 103 (11.3% [95% CI, 9.4%-13.6]) at follow-up (Table). Rates of PTSD according to PCL–Civilian Version and *DSM-5* criterion scoring method can be found in eTable 3 in Supplement 1. Prevalence of probable mental illness stratified by officer vs nonofficer status and left service vs still serving status can be found in eTables 4 and 5 in Supplement 1.

### Any Combat Injury

For those injured, compared with being uninjured, we observed an increase in risk of reporting probable PTSD (ARR, 2.00 [95% CI, 1.39-3.00]). Similar increases were observed for CMD (ARR for generalized anxiety, 1.92 [95% CI, 1.40-2.95]; ARR for depression, 1.78 [95% CI, 1.37-2.44]) and MHM (RR, 2.05 [95% CI, 1.35-3.16]) (Figure 2).

**Figure 2.**
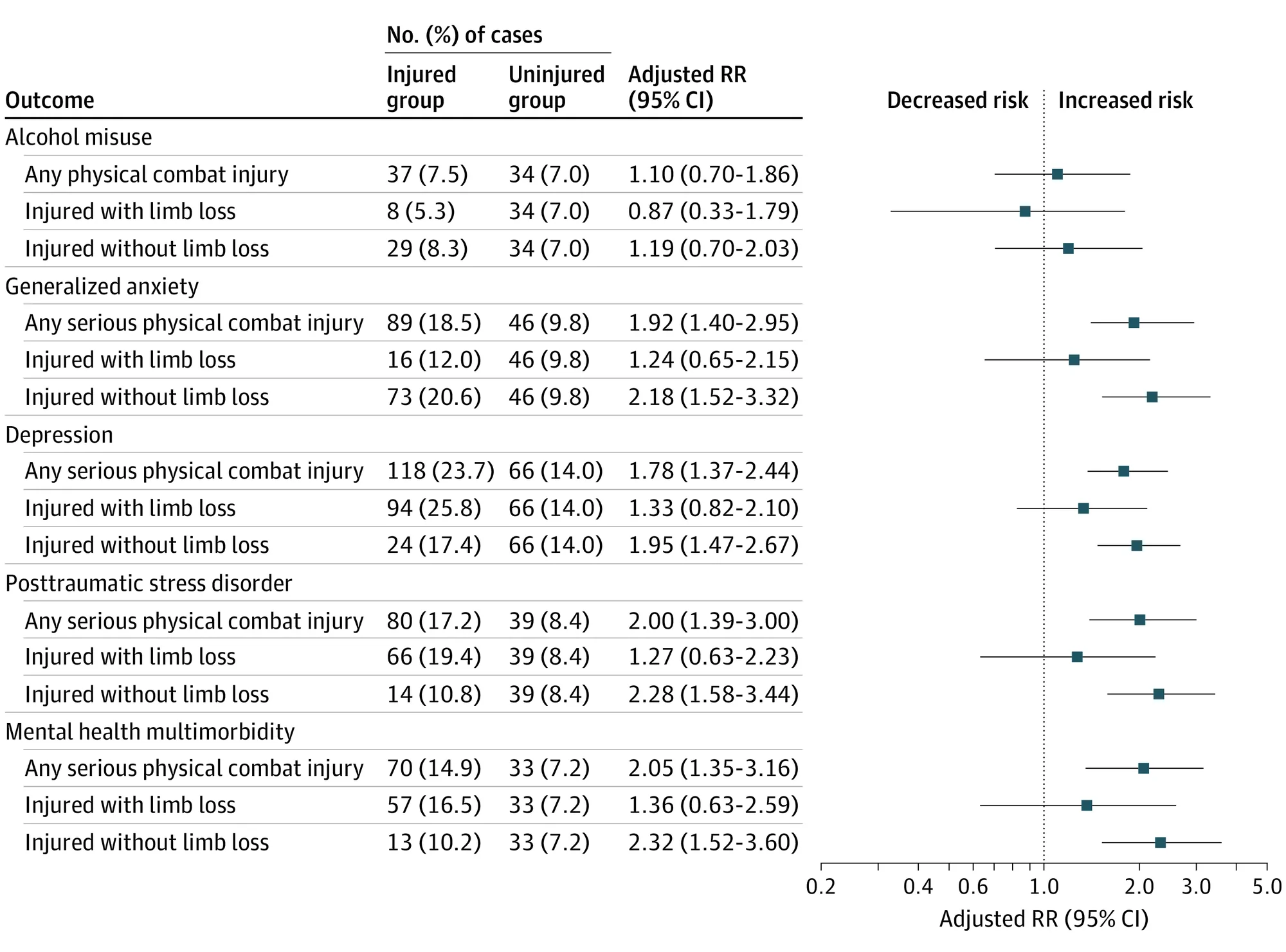
Dot Plot Showing Adjusted Risk Ratios (ARRs) From Bootstrapped General Linear Regression Models of Psychosocial Outcomes Stratified by Injury Status Model includes the confounding effect of socioeconomic status and age and excludes officers; bias-corrected 95% CIs are shown.

### Combat Injuries With or Without Associated Limb Loss

For those injured with associated limb loss, compared with being uninjured, we observed an increase in the risk of reporting probable PTSD, although this finding indicated uncertainty about the presence or magnitude of an association (ARR, 1.27 [95% CI, 0.63-2.23]). Similar risk profiles were present for CMD (ARR for generalized anxiety, 1.24 [95% CI, 0.65-2.15]; ARR for depression, 1.33 [95% CI, 0.82-2.10]) and MHM (ARR, 1.36 [95% CI, 0.63-2.59]) (Figure 2). For those injured without limb loss, compared with being uninjured, we observed an increase in risk of reporting probable PTSD (ARR, 2.28 [95% CI, 1.58-3.44]). Similar increases were observed for CMD (ARR for generalized anxiety, 2.18 [95% CI, 1.52-3.32]; ARR for depression, 1.95 [95% CI, 1.47-2.67]) and MHM (ARR 2.32 [95% CI, 1.52-3.60]) (Figure 2).

### Combat Injuries With and Without Deployment-Related TBI

For those injured with any deployment-related TBI, compared with being uninjured, we observed an increase in the risk of reporting probable PTSD (ARR, 2.39 [95% CI, 1.53-3.57]). Similar risk profiles were present for CMD (ARR for generalized anxiety, 2.19 [95% CI, 1.50-3.39]; ARR for depression, 2.04 [95% CI, 1.47-2.88]) and MHM (ARR, 2.52 [95% CI, 1.59-4.16]) (Figure 3). For those injured without any deployment-related TBI, compared with being uninjured, we observed an increase in the risk of reporting probable PTSD (ARR, 1.71 [95% CI, 1.10-2.68]). Similar risk was observed for CMD (ARR for generalized anxiety, 1.72 [95% CI, 1.18-2.73]; ARR for depression, 1.58 [95% CI, 1.15-2.25]); however, for MHM, the observed risk estimates indicated uncertainty about the presence or magnitude of an association (ARR, 1.69 [95% CI, 0.98-2.69]) (Figure 3).

**Figure 3.**
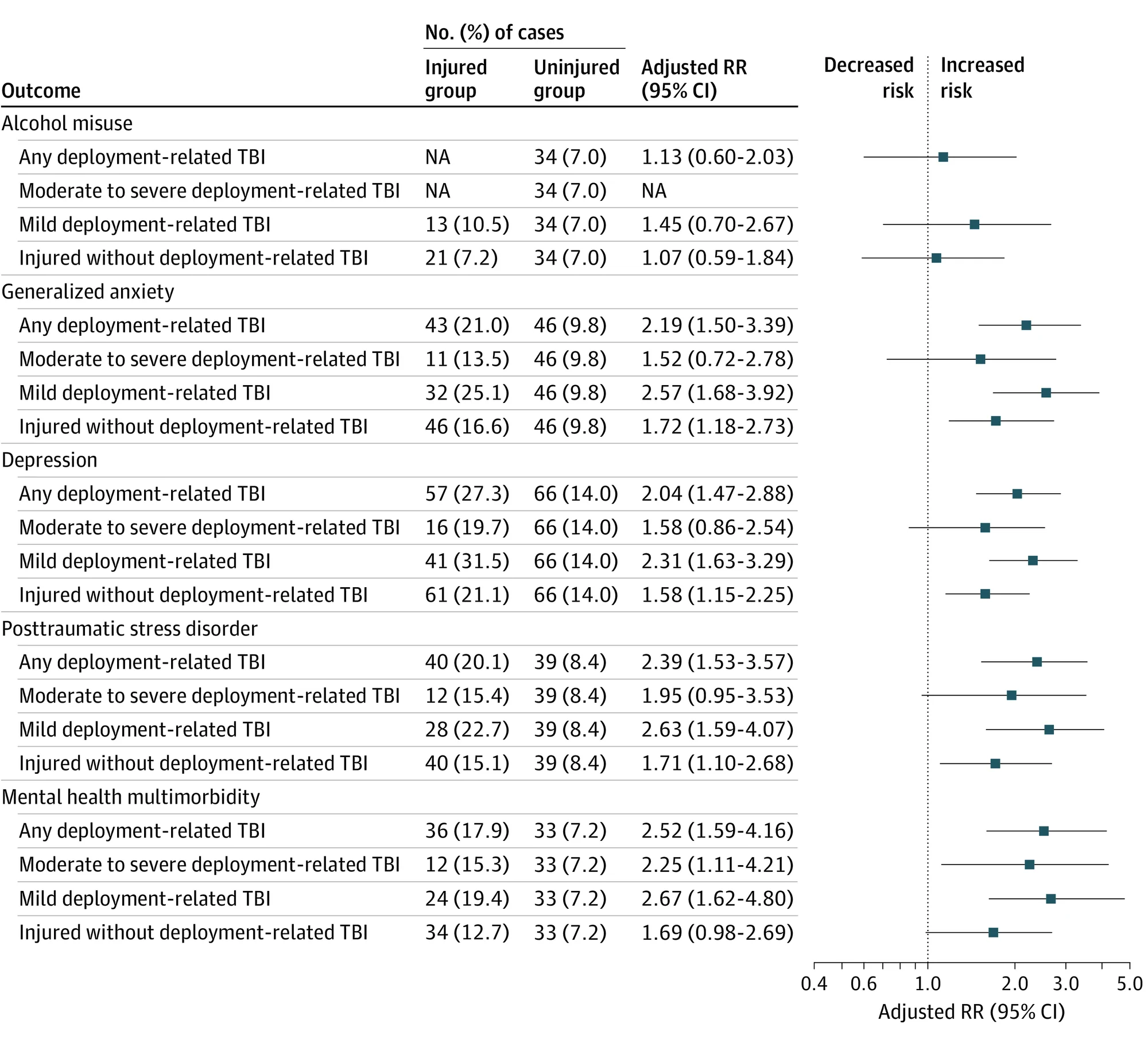
Dot Plot Showing Adjusted Risk Ratios (ARRs) From General Linear Model Regression of Psychosocial Outcomes, Stratified by Traumatic Brain Injury (TBI) Status Model includes the confounding effect of socioeconomic status and age and excludes officers; bias-corrected 95% CIs are shown. Deployment-related TBI group includes mild or moderate to severe TBI only. NA indicates that some data were suppressed to allow for confidentiality in line with Defence Statistics rounding policy.

### Combat Injuries With Mild or Moderate to Severe Deployment-Related TBI

For those injured with a mild TBI, compared with being uninjured, we observed an increase in risk of reporting probable PTSD (ARR 2.63 [95% CI, 1.59-4.07]). Similar increased risk was observed for CMD (ARR for generalized anxiety, 2.57 [95% CI, 1.68-3.92]; ARR for depression, 2.31 [95% CI, 1.63-3.29]) and MHM (ARR, 2.67 [95% CI, 1.62-4.80]) (Figure 3). For those injured with moderate to severe TBI, compared with being uninjured, we observed an increase in risk of reporting probable PTSD, although this finding indicated uncertainty about the presence or magnitude of an association (ARR, 1.95 [95% CI, 0.95-3.53]). Similar risk patterns were observed for CMD (ARR for generalized anxiety, 1.52 [95% CI, 0.72-2.78]; ARR for depression, 1.58 [95% CI, 0.86-2.54]), although we observed an increase in risk for reporting MHM (ARR, 2.25 [95% CI, 1.11-4.21]) (Figure 3).

### Combat Injuries With or Without Associated Limb Loss and Deployment-Related TBI

For those injured with associated limb loss and deployment-related TBI, compared with being uninjured, we observed an increase in risk of reporting probable PTSD, although this finding indicated uncertainty about the presence or magnitude of an association (ARR, 1.48 [95% CI, 0.64-2.84]). Similar risk profiles were observed for CMD (ARR for generalized anxiety, 1.41 [95% CI, 0.64-2.72]; ARR for depression, 1.57 [95% CI, 0.87-2.67]) and MHM (ARR 1.91 [95% CI, 0.79-3.66]) (Figure 4). For those injured with limb loss but without deployment-related TBI, compared to being uninjured, we observed an increase in risk of reporting probable PTSD, although this finding indicated uncertainty about the presence or magnitude of an association (ARR, 1.06 [95% CI, 0.38-2.26]). Similar risk profiles were observed for CMD (ARR for generalized anxiety, 1.07 [95% CI, 0.39-2.20]; ARR for depression, 1.10 [95% CI, 0.53-2.04]). For those injured with deployment-related TBI and without limb loss, compared with being uninjured, we observed an increase in risk of reporting probable PTSD (ARR, 2.80 [95% CI, 1.76-4.28]). Similar risk profiles were observed for CMD (ARR for generalized anxiety, 2.55 [95% CI, 1.68-4.05]; ARR for depression, 2.26 [95% CI, 1.62-3.21]) and MHM (ARR 2.81 [95% CI, 1.70-4.76]) (Figure 4). For those injured without limb loss and without deployment-related TBI, compared with being uninjured, we observed an increase in risk of reporting probable PTSD (ARR, 1.93 [95% CI, 1.20-3.11]). Similar risk profiles were observed for CMD (ARR for generalized anxiety, 1.92 [95% CI, 1.28-3.05]; ARR for depression, 1.74 [95% CI, 1.21-2.44]) and MHM (ARR, 1.98 [95% CI, 1.17-3.29]).

**Figure 4.**
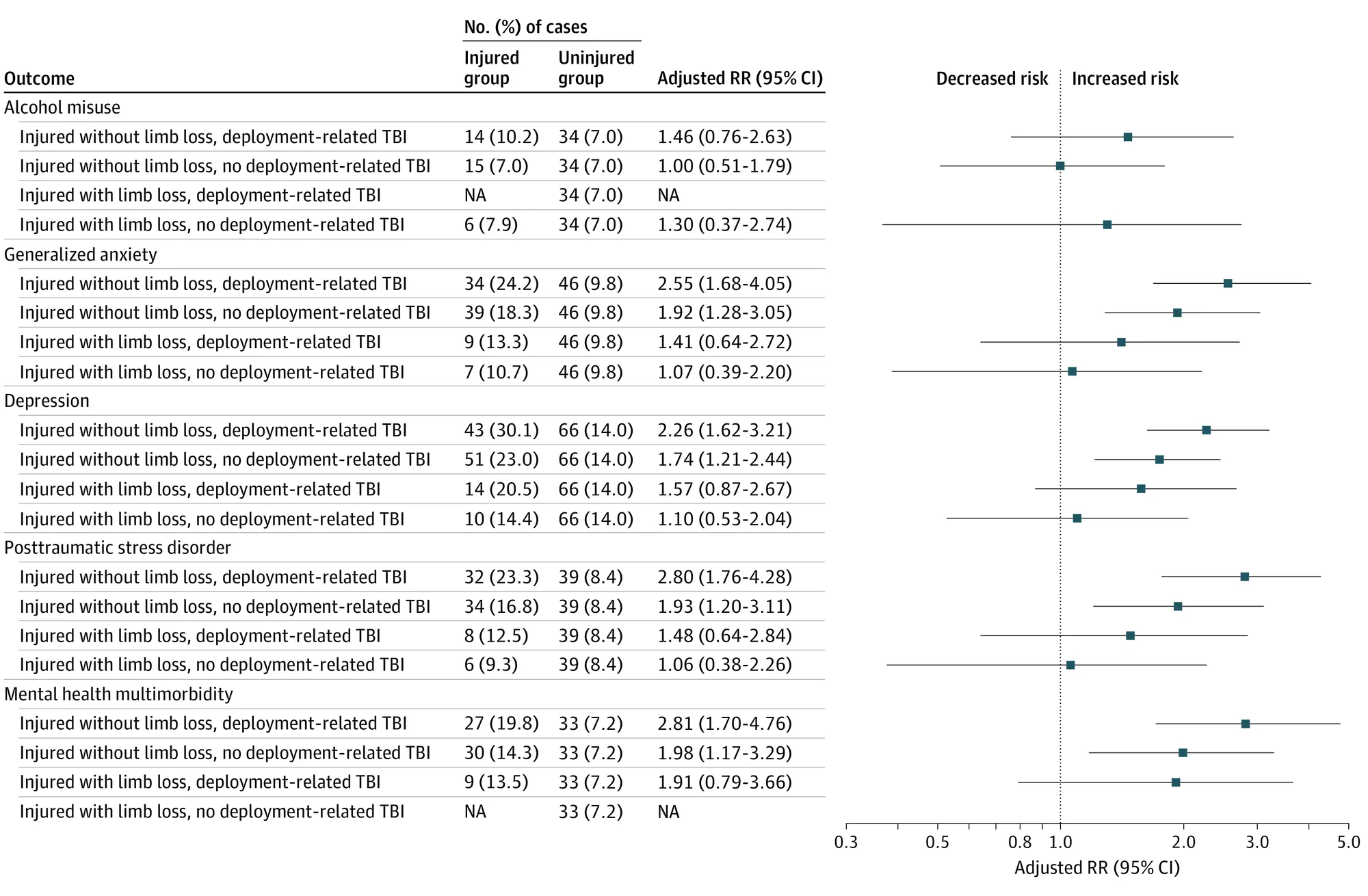
Dot Plot Showing Adjusted Risk Ratios (RRs) From General Linear Model Regression of Psychosocial Outcomes, Stratified by Injury Status Model includes confounding effect of socioeconomic status and age and excludes officers; bias-corrected 95% CIs are shown. Deployment-related traumatic brain injury (TBI) group includes mild or moderate to severe TBI only. NA indicates that some data were suppressed to allow for confidentiality in line with Defence Statistics rounding policy.

### Alcohol Misuse

Probable alcohol misuse was reported by 71 participants (7.3% [95% CI, 7.3%-9.2%) (Table). Relatively small risk estimates with wide 95% CIs (ARR range, 0.87 [95% CI, 0.33-1.79] to 1.46 [95% CI, 0.76-2.63]) indicate uncertainty regarding both the presence and magnitude of an association from sustaining any type of injury investigated as part of this analysis compared to the uninjured group (Figures 2-4).

## Discussion

In this cohort of UK military personnel who sustained physical combat injuries (injured group) in Afghanistan and a frequency-matched, demographically similar comparison group (uninjured group), we hypothesized that the injured group would have greater risk of reporting poor psychosocial outcomes compared with the uninjured group. We found partial support for this hypothesis. Sustaining a physical combat injury resulted in an elevation of risk for reporting probable CMD, PTSD, and MHM compared with the uninjured group. However, we observed divergence in the associated risk of reporting poor mental health outcomes depending on the injury profile present. Injuries without associated limb loss were associated with elevated risk of all mental health outcomes compared with the uninjured group, whereas injuries with associated limb loss had at most only small increases in risk, with 95% CIs indicating uncertainty in the presence and magnitude of this risk. Injuries with deployment-related mild and moderate to severe TBI were associated with elevated risk of all mental health outcomes, although 95% CIs for those with moderate to severe TBI indicated uncertainty in the presence and magnitude of this risk. We observed increased risk of reporting poor mental health outcomes in those injured with and without associated limb loss when co-occurring TBI was present. ARRs for reporting probable alcohol misuse between the injured and uninjured groups were small or inconclusive across all injury profiles.

Results of this study alongside results from the baseline assessment of the ADVANCE cohort^5^ show that the mental health outcomes of personnel injured with associated limb loss are similar to those of the uninjured group, especially when no deployment-related TBI is present (Figure 4), with analogous results observed for pain and suicidal ideation outcomes.^9,23^ These results differ from what has been observed among US military personnel, for whom limb amputation injuries are associated with elevated rates of poor mental health outcomes.^24^ Instead, we observed elevated risk of poor mental health outcomes in those injured without associated limb loss, which alongside baseline findings surrounding pain, suicidal ideation, and posttraumatic growth, indicate a negative psychological profile in this group.^5,9,23,25^ The fact that those injured without associated limb loss continued to have increased risk for all mental health outcomes even when deployment-related TBI was not present is particularly striking (Figure 4). It is possible that a combination of biopsychosocial factors are responsible for this increased risk. This group may underutilize available charitable and therapeutic services due to perceiving their injuries as comparatively less legitimate or deserving and from concern that their use of services could divert scarce resources from individuals with more overt or severe injuries,^26^ although no known research has investigated this phenomenon.

The risk of reporting poor mental health outcomes was elevated in personnel with deployment-related TBI compared with the uninjured group, especially in the case of mild TBI (Figure 3), which is in line with prior research.^7^ Moderate to severe TBI was also associated with elevated risk, but 95% CIs indicated uncertainty about the presence or magnitude of any risk from such an injury. Several things should be considered when interpreting these findings. First, personnel injured without evidence of deployment-related TBI still presented with increased risk of poor mental health outcomes (Figures 3 and 4). It is possible that varying clinical characteristics of TBI are responsible for different psychiatric presentations and contribute to any observed uncertainty. For example, the experience of posttraumatic amnesia for more than 24 hours, an indicator of moderate to severe TBI,^8^ may reduce the likelihood of presenting with PTSD symptoms.^27^ Furthermore, assessing symptomology of PTSD in individuals with TBI is difficult due to similarities in clinical presentation, structural brain changes, and endocrine and neurochemical dysfunction.^28^ Symptoms such as insomnia, emotional numbness, and exaggerated startle response are typical of both PTSD and TBI, and as such may lead to erroneous conclusions about the presence of PTSD or TBI. In the case of severe TBI, cognitive impairment may compromise the accuracy of self-report questionnaires (C.R.Z.S., G.B., L.O., et al, unpublished data, September 2025).^29^ Overall, our results suggest that deployment-related TBI increases the associated risk of poor mental health outcomes in injured personnel, although the extent of this elevation may depend on clinical features of TBI severity.

All groups studied, whether injured or not, reported greater rates of probable PTSD compared with the UK general population.^30^ Clinicians are advised to enquire about serving in the military to help understand the wider context of an individual’s injury and associated risks of poor outcomes. Using multidisciplinary expertise available through health initiatives relevant to UK veterans such as Op Courage, Op Restore, and Op Valour may help mitigate those risks. Injuries from the ongoing conflict in Ukraine are characterized by blast wounds to the extremities, TBI, and spinal cord injuries that are similar to patterns observed in the ADVANCE cohort.^1^ While there are some differences in the experiences of injured military personnel between these populations, such as longer medical evacuation times in Ukraine, understanding the protective and risk factors for mental illness in the ADVANCE cohort may be crucial in reducing the risk of mental illness among injured personnel from Ukraine or other future conflicts.

### Strengths and Limitations

This study has several strengths. First, to our knowledge, it represents the largest investigation of psychosocial outcomes and physical combat injury from the military deployments to Afghanistan among UK military personnel, using well-matched injured and uninjured personnel from whom to derive robust estimates of injury-associated risks. This cohort has retained a high number of participants at follow-up (1053 [92.0%]). Use of research nurse-led interviews regarding TBI, alongside investigation of trauma registry records, medical histories, and magnetic resonance imaging neuroradiology reports give a rich and detailed history of TBI.

The study also has several limitations. Our regression analyses were limited to nonofficer personnel, as rates of poor mental outcomes in the officer rank group were very low (eTable 4 in Supplement 1). All outcomes were based on self-report data only. Leaving service is associated with unique effects on mental illness (eTable 5 in Supplement 1). These effects could not be accounted for in these analyses, as they mediate the relationship between combat injury and mental illness, although the mediating effect of leaving service has been investigated previously.^31^ It is important to note that because the group with moderate to severe TBI was relatively small, our results should be interpreted cautiously. Finally, we investigated deployment-related mild and moderate to severe TBI outcomes only; other aspects of TBI, such as repeated, cumulative exposure over the lifetime, nondeployment TBI events, symptomatic (possible) TBI, or specific TBI clinical characteristics (eg, posttraumatic amnesia) were not investigated.

## Conclusions

In this cohort study of UK military personnel who sustained physical combat injuries in Afghanistan, we observed that noncommissioned officers and other ranks who sustained combat injuries had elevated risk of reporting probable CMD, PTSD, and MHM, but not alcohol misuse, compared with demographically similar personnel who sustained no such injuries. However, injury profiles were associated with heterogeneous risks: the presence of mild TBI and injuries without associated limb loss conferred the highest risk of poor mental health outcomes, whereas injuries with associated limb amputation had the smallest associated risks. Both visible injuries such as limb loss and nonvisible injuries such as TBI should be considered jointly in physical combat injury research and military medical policy to identify individuals at elevated risk of poor mental health outcomes.

## Notes

Supplement 1. **eFigure.** Timeline **eTable 1.** Measure Information **eTable 2.** Traumatic Brain Injury Groups **eMethods.** Weight Procedures **eTable 3.** Posttraumatic Stress Disorder Rates According to PCL-Civilian Version and *DSM-5* Scoring Method **eTable 4.** Mental Health Outcomes Stratified by Injury Status and Officer Status **eTable 5.** Mental Health Outcomes Stratified by Injury Status and Serving Status

Supplement 2.
Data Sharing Statement
