# Supplementary material for: Combat Injury, Limb Loss, Traumatic Brain Injury, and Psychosocial Outcomes Among Military Personnel
Source: JAMA Netw Open. 2026 Oct 6;9(10):e2637592. doi: 10.1001/jamanetworkopen.2026.37592 (PMC13643579; doi:10.1001/jamanetworkopen.2026.37592)
Supplement: Supplementary file 1 [file jamanetwopen-e2637592-s001.pdf]

Supplementary Online Content

Dyball D, See CRZ, Bennett AN, et al. Physical combat injury and psychosocial outcomes among military personnel. *JAMA Netw Open.* 2026;9(10):e2637592. doi:10.1001/jamanetworkopen.2026.37592

eFigure. Timeline

eTable 1. Measure Information

eTable 2. Traumatic Brain Injury Groups

eMethods. Weight Procedures

eTable 3. Posttraumatic Stress Disorder Rates According to PCL-Civilian Version and *DSM-5* Scoring Method

eTable 4. Mental Health Outcomes Stratified by Injury Status and Officer Status

eTable 5. Mental Health Outcomes Stratified by Injury Status and Serving Status

This supplementary material has been provided by the authors to give readers additional information about their work.

© 2026 Dyball D et al. *JAMA Network Open*.

eFigure. Timeline

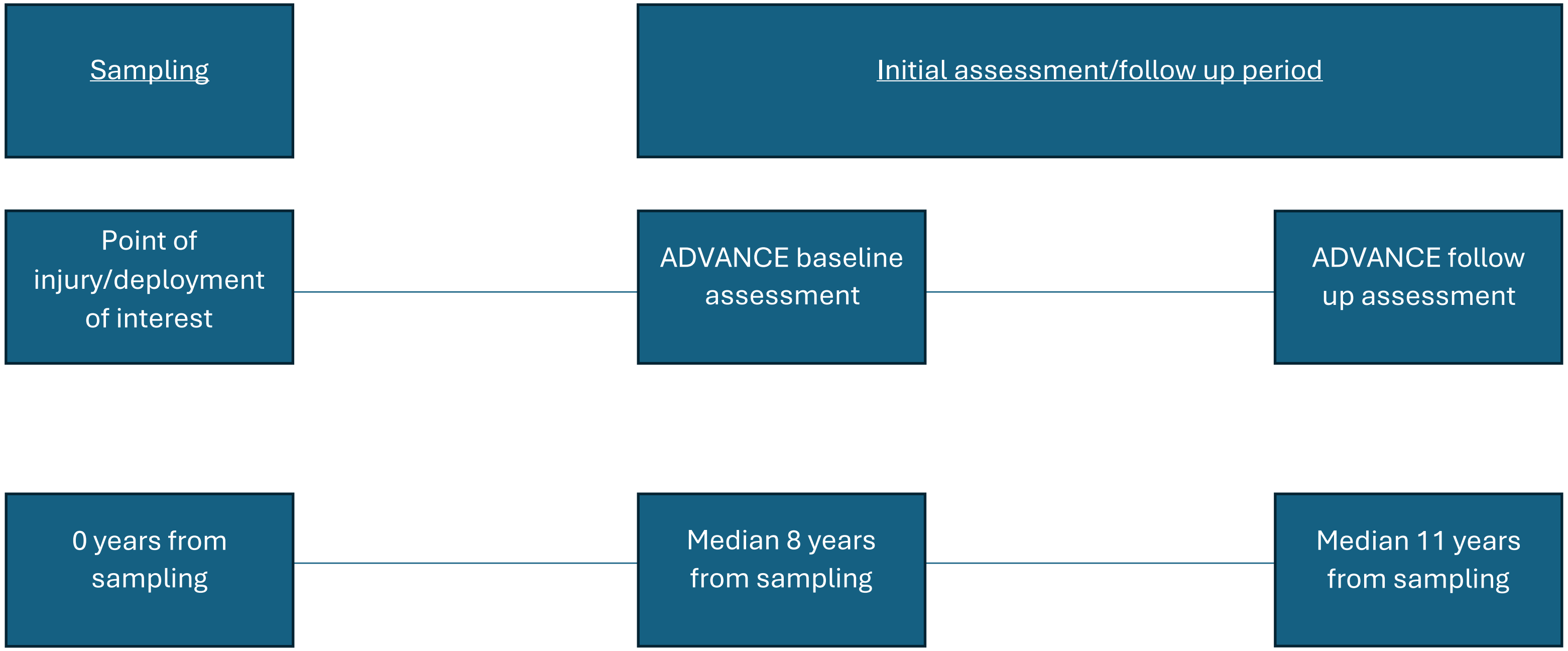

© 2026 Dyball D et al. *JAMA Network Open*.

eTable 1. Measure Information

Outcome	***Validation reference***	***Cut off score***
Alcohol misuse	*Saunders JB, Aasland OG, Babor TF, De La Fuente JR, Grant M. Development of the Alcohol Use Disorders Identification Test (AUDIT): WHO Collaborative Project on Early Detection of Persons with Harmful Alcohol Consumption-II. Addiction. 1993;88(6):791-804*	≥16
Generalised anxiety	*Spitzer RL, Kroenke K, Williams JB, Löwe B. A brief measure for assessing generalized anxiety disorder: the GAD-7. Archives of internal medicine. 2006;166(10):1092-7.*	≥10
Depression	*Kroenke K, Spitzer RL, Williams JB. The PHQ-9: validity of a brief depression severity measure. Journal of general internal medicine. 2001;16(9):606-13.*	≥10
PTSD	*Blevins CA, Weathers FW, Davis MT, Witte TK, Domino JL. The Posttraumatic Stress Disorder Checklist for DSM-5 (PCL-5): Development and Initial Psychometric Evaluation. J Trauma Stress. 2015;28(6):489-98.*	≥38
Mental health multimorbidity	*Nichter B, Norman S, Haller M, Pietrzak RH. Psychological burden of PTSD, depression, and their comorbidity in the US veteran population: suicidality, functioning, and service utilization. Journal of affective disorders. 2019;256:633-40.*	PTSD caseness plus generalised anxiety and/or depression

© 2026 Dyball D et al. *JAMA Network Open*.

eTable 2. Traumatic Brain Injury Groups

	Deployment-related TBI	Deployment-related TBI severity	Deployment-related TBI	
			With limb loss	Without limb loss
Uninjured group	0	0	0	0
Injured group without deployment-related TBI	1	1	3	1
Injured group with deployment-related symptomatic TBI	1	2	3	1
Injured group with deployment-related mild TBI	2	3	4	2
Injured group with deployment-related moderate-severe TBI	2	4	4	2

© 2026 Dyball D et al. *JAMA Network Open*.

eMethods. Weight Procedures

Sampling weights were applied to the injured group to account for the under-sampling of the less-seriously injured group (1, 2). Response weights were applied based on age, rank, and service at time of injury/deployment of interest to take into account that officers, Royal Marines, and slightly older participants were more represented in those who responded to recruitment at baseline assessment. An attrition weight for the first follow-up was calculated using a logistic regression model including data collected at baseline; LASSO was used to reduce the model and the final model included smoking status, age, combat role, rank, and relationship status. A longitudinal weight for the first follow-up was calculated by multiplying the attrition weight at the first follow-up by the overall baseline weight (2).

1. Dyball D, Bennett AN, Schofield S, Cullinan P, Boos CJ, Bull AM, et al. Mental health outcomes of male UK military personnel deployed to Afghanistan and the role of combat injury: analysis of baseline data from the ADVANCE cohort study. The Lancet Psychiatry. 2022;9(7):547-54

2. Dyball D, Schofield S, Burdett H, Boos CJ, Bull AMJ, Cullinan P, et al. Evaluating the physical and psychosocial impact of serious physical combat injuries in UK armed forces personnel-the ADVANCE cohort study. European Journal of Epidemiology. 2025.

© 2026 Dyball D et al. *JAMA Network Open*.

eTable 3. Posttraumatic Stress Disorder Rates According to PCL-Civilian Version and *DSM-5* Scoring Method

	Uninjured group	All combat injuries	Limb loss injuries		Deployment-related TBI by severity			
	**Overall group(n=524)**	**Overall group (n=523)**	**Non-amputation injury subgroup (n=382)**	**Amputation injury subgroup (n=141)**	**No TBI (n=218)**	**Symptomatic TBI (n=80)**	**Mild TBI (n=144)**	**Moderate/Severe TBI (n=81)**
**Post-Traumatic Stress Disorder (PCL-C ≥50)**	38 (8·2; 6·0, 11·2)	81 (17·2; 14·0, 21·0)	67 (19·4; 15·5, 24·0)	14 (10·8; 6·4, 17·6)	31 (15·8; 11·2, 21·7)	8 (11·9; 5·8, 23·0)	29 (23·1; 16·4, 31·4)	13 (16·1; 9·4, 26·4)
**Post-Traumatic Stress Disorder (PCL-5 DSM5*)**	51 (11.2; 8.6, 14.5)	83 (17.6; 14.3, 21.4)	68 (19.8; 15.8, 24.4)	15 (11.0; 6.6, 17.7)	32 (16.3; 11.7, 22.4)	10 (14.4; 7.6, 25.6)	29 (22.8; 16.2, 31.1)	12 (14.7; 8.3, 24.8)

*Defined as at least one symptom of moderate or greater severity in criterion B (item1-5) and criterion C (item 6-7) and two symptoms of moderate or greater severity in criterion D (item 8-14) and criterion E (item 15-20)

© 2026 Dyball D et al. *JAMA Network Open*.

eTable 4. Mental Health Outcomes Stratified by Injury Status and Officer Status

	Uninjured group		All combat injuries		Limb loss injuries				Deployment-related TBI by severity							
	Overall group(n=524)		Overall group (n=523)		Non-amputation injury subgroup (n=382)		Amputation injury subgroup (n=141)		No TBI (n=218)		Symptomatic TBI (n=80)		Mild TBI (n=144)		Moderate/Severe TBI (n=81)	
	Officer (n=74)	Non-officer (n=450)	Officer (n=57)	Non officer (n=466)	Officer (n=45)	Non officer (n=337)	Officer (n=12)	Non officer (n=129)	Officer (n=24)	Non officer (n=194))	Officer (n=10)	Non officer (n=70)	Officer (n=15)	Non officer (n=129)	Officer (n=8)	Non officer (n=73)
**Mental health outcomes No. (%)**																
Alcohol misuse (AUDIT ≥16)	~	31 (7.3; 5.2, 10.3)	~	36 (7.8; 5.7, 10.9)	~	28 (8.7; 6.0, 12.4)	~	8 (5.6; 2.8, 11.0)	~	15 (7.9; 4.7, 13.0)	~	5 (6.0; 2.5, 14.3)	~	13 (11.2; 6.5, 18.6)	~	~
Anxiety (GAD7 ≥10)	~	43 (10.4; 7.7, 13.8)	~	87 (19.5; 16.0, 23.6)	~	71 (21.8; 17.5, 26.8)	0 (0,0)	16 (12.7; 7.8, 20.0)	~	37 (19.8; 14.6, 26.3)	0 (0,0)	7 (10.7; 4.9, 24.9)	0 (0,0)	32 (26.7; 19.4, 35.6)	0 (0,0)	11 (14.3; 7.8, 24.6)
Depression (PHQ9 ≥10)	6 (8.6; 3.8, 18.2)	60 (14.5; 11.3, 18.3)	5 (9.9; 4.0, 22.1)	113 (24.7; 20.8, 29.0)	5 (12.2; 5.0, 27.0)	89 *26.8; 22.2, 32.0)	0 (0,0)	24 (18.3; 12.4, 26.2)	~	47 (24.1; 18.4, 30.9)	~	10 (14.9; 7.8, 26.7)	~	40 (33.0; 25.0, 42.1)	0 (0,0)	16 (20.8; 12.8, 32.0)
Post-Traumatic Stress Disorder (PCL-5 ≥38)	~	37 (8.9; 6.5, 12.2)	~	78 (18.1; 14.7, 22.1)	~	64 (20.4; 16.2, 25.4)	0 (0,0)	14 (11.4; 6.7, 18.6)	~	30 (16.7; 11.9, 23.1)	~	8 (13.0; 6.3, 24.8)	0 (0,0)	28 (24.1; 17.1, 33.0)	0 (0,0)	12 (16.2; 9.2, 27.1)
Mental health multimorbidity (caseness on PCL-5 & PHQ9 or GAD7)	~	31 (7.6; 5.4, 10.7)	~	68 (15.6; 12.4, 19.4)	~	55 (17.3; 13.4, 22.0)	0 (0,0)	13 (10.7; 6.2, 17.9)	~	28 (15.2; 10.6, 21.3)	~	~	0 (0,0)	24 (20.7; 14.1, 29.2)	0 (0,0)	12 (16.1; 9.1, 26.8)

© 2026 Dyball D et al. *JAMA Network Open*.

	Uninjured group		All combat injuries		Limb loss injuries				Deployment-related TBI by severity							
	Overall group(n=524)		Overall group (n=523)		Non-amputation injury subgroup (n=382)		Amputation injury subgroup (n=141)		No TBI (n=218)		Symptomatic TBI (n=80)		Mild TBI (n=144)		Moderate/Severe TBI (n=81)	
	Officer (n=74)	Non-officer (n=450)	Officer (n=57)	Non officer (n=466)	Officer (n=45)	Non officer (n=337)	Officer (n=12)	Non officer (n=129)	Officer (n=24)	Non officer (n=194))	Officer (n=10)	Non officer (n=70)	Officer (n=15)	Non officer (n=129)	Officer (n=8)	Non officer (n=73)

~Some data suppressed to allow for confidentiality in line with Defence Statistics rounding policy (https://www.gov.uk/government/publications/defence-statistics-policies/ministry-of-defence-disclosure-control-and-rounding-policy).

AUDIT Alcohol Use Disorder Identification Toolkit GAD7 Generalized Anxiety Disorder-7 PCL-5 Post-traumatic stress disorder checklist diagnostics statistical manual 5 PHQ9 Patient Health Questionnaire-9 PTSD Post-Traumatic Stress Disorder

eTable 5. Mental Health Outcomes Stratified by Injury Status and Serving Status

	Uninjured group		All combat injuries		Limb loss injuries				Deployment-related TBI by severity							
	Overall group(n=524)		Overall group (n=523)		Non-amputation injury subgroup (n=382)		Amputation injury subgroup (n=141)		No TBI (n=218)		Symptomatic TBI (n=80)		Mild TBI (n=144)		Moderate/Severe TBI (n=81)	
	In-service (n=356)	Left service (n=168)	In-service (n=109)	Left service (n=414)	In-service (n=106)	Left service (n= 276)	In-service (n<5 ~)	Left service (n>136 ~)	In-service (n=64)	Left service (n=154)	In-service (n=11)	Left service (n=69)	In-service (n=25)	Left service (n=119)	In-service (n=9)	Left service (n=72)
**Mental health outcomes No. (%)**																
Alcohol misuse (AUDIT ≥16)	20 (5.8; 3.7, 9.0)	10 (12.5; 6.6, 22.3)	10 (9.4; 5.0, 17.1)	26 (7.4 (5.0, 10.8)	10 (9.7; 5.2, 17.6)	19 (8.5; 5.4, 13.1)	0 (0,0)	7 (5.1; 2.4, 10.5	7 (11.3; 5.2, 22.6)	8 (6.3; 3.0, 12.5)	~	~	~	11 (12.1; 6.7, 20.8)	0 (0,0)	~

© 2026 Dyball D et al. *JAMA Network Open*.

	Uninjured group		All combat injuries		Limb loss injuries				Deployment-related TBI by severity							
	Overall group(n=524)		Overall group (n=523)		Non-amputation injury subgroup (n=382)		Amputation injury subgroup (n=141)		No TBI (n=218)		Symptomatic TBI (n=80)		Mild TBI (n=144)		Moderate/Severe TBI (n=81)	
	In-service (n=356)	Left service (n=168)	In-service (n=109)	Left service (n=414)	In-service (n=106)	Left service (n= 276)	In-service (n<5 ~)	Left service (n>136 ~)	In-service (n=64)	Left service (n=154)	In-service (n=11)	Left service (n=69)	In-service (n=25)	Left service (n=119)	In-service (n=9)	Left service (n=72)
Anxiety (GAD7 ≥10)	27 (8.1; 5.5, 11.6)	11 (14.5; 8.1, 24.8)	15 (13.9; 8.4, 22.3)	67 (19.6; 15.7, 24.3)	15 (14.4; 8.7, 23.0)	53 (23.3; 18.1, 29.4)	0 (0,0)	14 (11.7; 6.9, 19.1)	10 (14.5; 7.7, 25.7)	25 (19.8; 13.6, 28.0)	0 (0,0)	7 (12.0; 5.5, 24.2)	~	25 (26.2; 18.2, 36.1)	~	10 (15.8; 8.4, 27.7)
Depression (PHQ9 ≥10)	36 (11.0; 8.0, 15.0)	17 (22.2; 14.1, 33.2)	20 (17.5; 11.3, 26.2)	88 (24.9; 20.5, 29.8)	20 (18.1; 11.7, 28.0)	67 (28.7; 23.1, 35.0)	0 (0,0)	21 (16.8; 11.0, 24.7)	12 (17.1; 9.6, 28.6)	34 (25.4; 18.5, 33.8)	~	9 (13.9; 7.0, 26.0)	6 (25.3; 10.7, 48.8)	32 (33.4; 24.6, 43.6)	~	13 (20.6; 12.0, 33.1)
Post-Traumatic Stress Disorder (PCL-5 ≥38)	12 (3.5; 2.0, 6.2)	14 (19.0; 11.4, 29.9)	12 (11.1; 6.2, 19.0)	61 (18.7; 14.8, 23.5)	12 (11.5; 6.4, 19.6)	49 (22.6; 17.4, 28.8)	0 (0,0)	12 (10.4; 5.9, 17.7)	7 (10.9; 5.0, 22.2)	21 (17.4; 11.5, 25.4)	0 (0,0)	9 (15.5; 7.9, 28.3)	~	21 (23.7; 15.9, 33.8)	~	10 (16.6; 8.9, 28.9)
Mental health multimorbidity (caseness on PCL-5 & PHQ9 or GAD7)	10 (3.0; 1.6, 5.5)	12 (16.3; 9.4, 26.9)	9 (7.8; 4.0, 14.7)	53 (16.2; 12.5, 20.7)	9 (8.1; 4.1, 15.2)	42 (19.2; 14.4, 25.1)	0 (0,0)	11 (9.7; 5.4, 16.9)	6 (8.4; 3.6 18.2)	19 (15.6; 10.1, 23.4)	0 (0,0)	5 (9.2; 3.6, 21.5)	~	19 (21.2; 13.8, 31.0)	~	10 (16.4; 8.8, 28.6)

~Some data suppressed to allow for confidentiality in line with Defence Statistics rounding policy (https://www.gov.uk/government/publications/defence-statistics-policies/ministry-of-defence-disclosure-control-and-rounding-policy).

AUDIT Alcohol Use Disorder Identification Toolkit GAD7 Generalized Anxiety Disorder-7 PCL-5 Post-traumatic stress disorder checklist diagnostics statistical manual 5 PHQ9 Patient Health Questionnaire-9 PTSD Post-Traumatic Stress Disorder

© 2026 Dyball D et al. *JAMA Network Open*.
