# Supplementary material for: Combat Injury, Limb Loss, Traumatic Brain Injury, and Psychosocial Outcomes Among Military Personnel
Source: JAMA Netw Open. 2026 Oct 6;9(10):e2637592. doi: 10.1001/jamanetworkopen.2026.37592 (PMC13643579; doi:10.1001/jamanetworkopen.2026.37592)
Supplement: Supplementary file 2 [file jamanetwopen-e2637592-s002.pdf]

Data Sharing Statement

Dyball. Combat Injury, Limb Loss, Traumatic Brain Injury, and Psychosocial Outcomes Among Military Personnel. *JAMA Netw Open*. Published October 06, 2026.
doi:10.1001/jamanetworkopen.2026.37592

Data
Data available: No

Additional Information
Explanation for why data not available: Because of the sensitive nature of the data collected for ADVANCE, its data cannot be made publicly available, but requests to access the dataset from qualified researchers trained in human subject confidentiality may be sent to adv_data_team@imperial.ac.uk and will be considered by the Project Board on a case-by-case basis, and subject to UK Ministry of Defence clearance. Details of the study data are available on the ADVANCE website (https://www.advancestudydmrc.org.uk/research-portal/).
